# Emotional Responses to AI-Powered Personalised Advertising: The Role of Perceived Empathy and Social Cognition in Consumer Decision-Making

**DOI:** 10.3390/jintelligence14060098

**Published:** 2026-06-03

**Authors:** Cristian Ionuţ Tatu, Raluca-Giorgiana Chivu (Popa), Mihai Cristian Orzan, Daniel Moise, Larisa Boboc (Dumitru)

**Affiliations:** Faculty of Marketing, Bucharest University of Economic Studies, 010404 Bucharest, Romania; cristitatu@mk.ase.ro (C.I.T.); mihai.orzan@ase.ro (M.C.O.); moisedaniel@mk.ase.ro (D.M.); larisa.boboc@mk.ase.ro (L.B.)

**Keywords:** AI-powered advertising, perceived AI empathy, social cognition, trust in AI, emotional arousal, cognitive elaboration, consumer engagement, purchase intention, PLS-SEM, personalisation

## Abstract

The rapid proliferation of artificial intelligence (AI) in digital advertising has fundamentally transformed how brands communicate with consumers, shifting from generic mass messaging toward highly personalised, emotionally targeted experiences. Despite growing interest in AI-driven marketing, limited empirical research has examined how consumers’ socio-emotional processing mechanisms, particularly perceived empathy and social cognition, mediate the relationship between AI-powered ad personalisation and downstream consumer decision-making outcomes. This study addresses this gap by investigating the emotional and cognitive responses triggered by AI-personalised advertising among Romanian consumers. Using a quantitative survey design, data were collected from a sample of 234 adult respondents (18–65 years) in Romania, broadly aligned with key Romanian demographic distributions across age, gender, and residential area. Structural equation modelling using the Partial Least Squares (PLS-SEM) approach was employed to test the proposed conceptual model, which integrates constructs of AI-powered ad personalisation, trust in AI, perceived AI empathy, emotional arousal, cognitive elaboration, social cognition, consumer engagement, and purchase intention. The results reveal that perceived empathy toward AI-generated advertising positively influences emotional arousal and cognitive elaboration, which in turn significantly predict consumer engagement and purchase intention. Trust in AI emerged as a critical sequential mediator, while social cognition moderated the personalisation-to-trust pathway. The study yields a validated marketing model that captures the socio-emotional dynamics underlying consumer responses to AI advertising. These findings contribute to the theoretical understanding of human–AI interaction through a social cognition and emotions lens, while offering practical implications for the design of emotionally intelligent, AI-driven advertising strategies. Limitations and future research directions are discussed.

## 1. Introduction

Artificial intelligence is reshaping the global advertising landscape at an accelerating pace. By 2025, global spending on AI-enabled marketing technologies had surpassed USD 107 billion (Statista, 2024), and predictive personalisation engines now underpin the majority of programmatic advertising ecosystems. This technological transition marks a qualitative shift in the consumer–advertising encounter: where traditional campaigns broadcast uniform messages to heterogeneous audiences, AI-powered systems construct individualised advertising experiences that adapt in real time to each consumer’s behavioural history, contextual signals, and inferred preferences (Davenport et al., 2020; Haenlein & Kaplan, 2019).

Despite the commercial ubiquity of AI-personalised advertising, its psychological underpinnings remain incompletely understood. The extant literature has predominantly examined personalisation through utilitarian or cognitive lenses, focusing on relevance perceptions, privacy trade-offs, and attitudinal outcomes (Aguirre et al., 2015; Bleier & Eisenbeiss, 2015), while the affective and socio-cognitive dimensions of consumer responses have received comparatively limited scholarly attention (Tran, 2017). This is a significant gap, given that emotional processing and social cognition are now widely recognised as central determinants of consumer behaviour across digital contexts (Adolphs, 2009; Holbrook & Batra, 1987).

The present study is motivated by a theoretical lacuna at the intersection of three converging research streams: (1) the psychology of human–AI interaction and the conditions under which consumers attribute social and emotional qualities to AI agents (Shank et al., 2019; Glikson & Woolley, 2020); (2) the role of socio-emotional skills, including empathy, emotional regulation, and social cognition, in shaping information processing and decision-making (Decety & Jackson, 2004; Frith & Frith, 2003); and (3) the consumer behaviour consequences of AI-driven advertising personalisation, with particular attention to trust formation and engagement (Kumar et al., 2010; Bucea-Manea-Tonis et al., 2026). These streams have developed largely in isolation; the present study seeks to integrate them within a unified empirical model.

We propose and test a sequential mediation model in which AI-powered ad personalisation builds consumer trust in the AI system, which in turn activates perceived AI empathy, the consumer’s subjective experience that the AI agent understands and resonates with their emotional and situational needs. Perceived AI empathy then bifurcates into two parallel mediating pathways: emotional arousal and cognitive elaboration. Both pathways converge on consumer engagement, which ultimately predicts purchase intention. The individual-level moderating role of social cognition is examined at the personalisation-to-trust link, reflecting the hypothesis that socially cognitively sophisticated consumers are more apt to interpret AI personalisation through a relational schema.

The study makes four principal contributions to the literature. First, it introduces perceived AI empathy as a theoretically grounded mediating construct that bridges the cognitive-trust domain and the affective-processing domain in AI advertising research. Second, it positions social cognition, a construct central to the focus of this Special Issue, as a meaningful individual-difference boundary condition within an advertising effectiveness model, thereby extending its explanatory reach beyond developmental and clinical contexts into consumer behaviour. Third, it proposes consumer engagement as the integrative mechanism through which both affective and cognitive pathways jointly determine purchase outcomes, offering a more complete account of the advertising effectiveness process than single-pathway models. Fourth, the study contributes empirical evidence from Romania, an underrepresented Central and Eastern European (CEE) digital market context, thereby extending the geographic and cultural scope of the human–AI interaction literature.

What distinguishes this study from prior work on AI personalisation (Aguirre et al., 2015; Tam & Ho, 2005), trust in AI (Glikson & Woolley, 2020; Araujo et al., 2020), and perceived empathy in digital contexts (Zhou et al., 2020; Xu & Chan-Olmsted, 2022) is the integration of these constructs into a unified sequential mediation model that explicitly captures the asymmetric dual-pathway architecture connecting perceived AI empathy to consumer engagement. No prior study has jointly modelled perceived AI empathy as a mediating hub, social cognition as a moderating boundary condition, and consumer engagement as the integrative outcome mechanism within a single empirical framework. This specificity of theoretical focus constitutes the principal novelty of the present contribution.

The empirical base consists of a cross-sectional survey administered to a quota-sampled consumer panel of 234 Romanian adult consumers, broadly reflective of key national demographic distributions (ages 18–65). Structural equation modelling using the Partial Least Squares approach (PLS-SEM) was employed to estimate and test the proposed model. The choice of PLS-SEM is justified by the exploratory–predictive nature of the study, the inclusion of newly operationalized constructs, and the moderate sample size (Hair et al., 2019; Ringle et al., 2020).

The remainder of the paper is structured as follows. Section 2 reviews the theoretical background and develops the study hypotheses. Section 3 presents the research design and methodology. Section 4 reports the measurement model and structural model results. Section 5 discusses the findings in light of theoretical and managerial implications. Section 6 concludes with limitations and directions for future research.

## 2. Theoretical Background and Hypothesis Development

### 2.1. AI-Powered Personalised Advertising: Conceptual Definition and Theoretical Framework

The emergence of artificial intelligence in digital marketing has fundamentally reconfigured the logic of advertising communication, enabling unprecedented levels of message personalisation at scale. AI-powered personalised advertising refers to the use of machine learning algorithms, natural language processing, and behavioural data analytics to dynamically tailor advertising content, timing, and format to individual consumer profiles (Choi et al., 2020; Radu et al., 2017). Unlike traditional one-to-many advertising approaches, AI-driven systems continuously learn from user interactions, refining recommendations and creative executions in real time (Davenport et al., 2020).

For the purposes of this study, AI-powered ad personalisation (AIPA) is operationalized as a unidimensional reflective construct capturing the consumer’s subjective perception that digital advertising directed at them is individually tailored (in terms of content, timing, and format) through the application of machine learning algorithms and behavioural data analytics. This construct functions as the primary exogenous stimulus in the proposed model, reflecting the consumer-facing output of AI personalisation technologies rather than the underlying technical mechanism per se.

**Figure 1:**
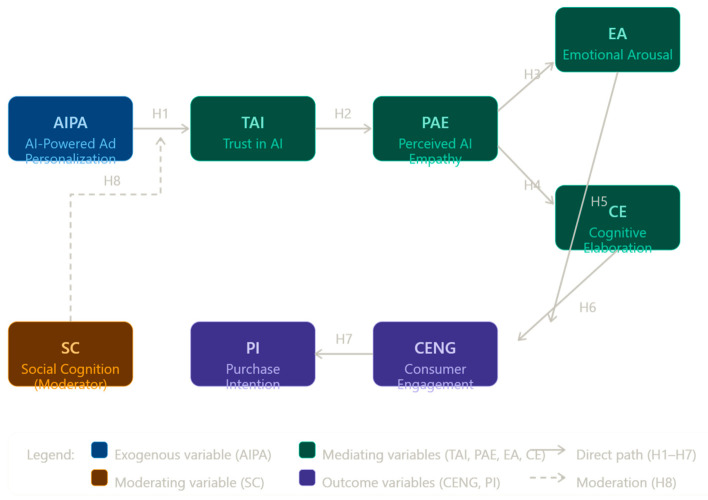
Proposed conceptual model of emotional responses to AI-powered personalised advertising. Solid arrows denote direct hypothesised relationships (H1–H7); dashed arrow denotes moderation (H8). AIPA = AI-Powered Ad Personalisation; TAI = Trust in AI; PAE = Perceived AI Empathy; EA = Emotional Arousal; CE = Cognitive Elaboration; SC = Social Cognition; CENG = Consumer Engagement; PI = Purchase Intention.

Several theoretical frameworks have been mobilised to explain consumer responses to personalised advertising. The *Uses and Gratifications Theory* posits that individuals actively select media content that satisfies specific cognitive and affective needs (Katz et al., 1973); AI personalisation, by anticipating these needs, increases the likelihood of perceived relevance and positive evaluative responses. Similarly, the *Stimulus–Organism–Response (S-O-R) framework* (Mehrabian & Russell, 1974) conceptualises AI-generated ad stimuli as environmental inputs that trigger internal psychological states, including emotional and cognitive appraisals, which ultimately mediate behavioural responses such as purchase intention. This study adopts the S-O-R perspective as the overarching theoretical lens, positioning AI-powered ad personalisation as the key stimulus that activates a cascade of mediating constructs culminating in consumer decision-making outcomes (Jacoby, 2002). Within the S-O-R framework adopted here, the three components are mapped as follows: the Stimulus is AI-powered ad personalisation (AIPA), representing the algorithmically tailored advertising input to which the consumer is exposed; the Organism components are the internal psychological states activated by this stimulus, comprising Trust in AI (TAI), Perceived AI Empathy (PAE), Emotional Arousal (EA), and Cognitive Elaboration (CE), moderated by Social Cognition (SC); and the Response outcomes are Consumer Engagement (CENG) and Purchase Intention (PI). Figure 1 illustrates this S-O-R architecture in full.

Empirical research has consistently demonstrated that perceived ad personalisation enhances attitudinal and behavioural outcomes, including ad liking, click-through rates, and conversion (Aguirre et al., 2015; Tam & Ho, 2005). However, the psychological mechanisms through which personalisation exerts its influence, specifically those involving socio-emotional processing, remain insufficiently theorised in the extant literature (Zeng et al., 2021). The present study seeks to address this gap by examining how AI personalisation generates trust and perceived empathy, and how these constructs in turn activate emotional and cognitive processes that drive engagement and purchase intention.

### 2.2. Trust in AI: From Personalisation to Perceived Empathy

Trust is a foundational construct in consumer–brand relationships and has received increasing scholarly attention in the context of human–AI interaction (Luhmann, 1979; Lee & See, 2004). In the proposed model, Trust in AI functions in a dual capacity: as the direct outcome of AI-powered ad personalisation (H1, a direct path) and as the sequential mediator that transmits the effect of personalisation to Perceived AI Empathy (H2). The section title reflects this mediating role within the broader sequential chain, rather than a claim that H1 itself is an indirect path. In the AI advertising context, trust in AI (TAI) refers to consumers’ generalised belief that AI systems will act reliably, competently, and in alignment with their interests (Glikson & Woolley, 2020). Building on McKnight et al.’s (2002) multidimensional conceptualization, scholars have identified capability-, benevolence-, and integrity-based dimensions as particularly relevant when consumers interact with algorithmic systems.

The relationship between AI-powered personalisation and trust is theoretically grounded in the *Information Processing Theory* (Miller, 1956) and the *Technology Acceptance Model (TAM)* (F. D. Davis, 1989): when consumers perceive that an AI system accurately infers and responds to their preferences, this serves as a competence signal that increases trust calibration (Komiak & Benbasat, 2006). Conversely, overly intrusive or inaccurate personalisation can trigger trust violations and privacy concerns (Orzan et al., 2014). Recent experimental evidence indicates that the quality and contextual relevance of AI-personalised content significantly predicts trust formation, even when consumers are aware of the algorithmic nature of the content (Araujo et al., 2020).

Trust functions as a cognitive gateway that lowers perceived risk and reduces the effort required to process subsequent advertising stimuli (D. J. Kim et al., 2008). In the model proposed here, Trust in AI is the first mediating link between the personalisation stimulus and the consumer’s perceived empathic quality of the AI agent, consistent with emerging theoretical perspectives on human–AI relational dynamics (Pentina et al., 2023; Choudhury & Shamszare, 2023).

**H1.**  *AI-powered ad personalisation is positively associated with consumer Trust in AI.*

### 2.3. Perceived AI Empathy

Empathy, the capacity to understand and vicariously share the emotional states of another, has traditionally been conceptualised as an exclusively human socio-cognitive faculty (Decety & Jackson, 2004). However, the proliferation of conversational AI, emotionally adaptive interfaces, and affective computing has prompted scholars to investigate whether consumers can perceive empathic qualities in AI-generated communication (Johanson et al., 2023; Zhou et al., 2020). *Perceived AI empathy (PAE)* is defined here as the consumer’s subjective assessment that an AI system understands, resonates with, and appropriately responds to their emotional and situational needs (Go & Sundar, 2019).

The theoretical basis for PAE lies at the intersection of the *Computers Are Social Actors (CASA) paradigm* (Nass et al., 1994) and *Affective Computing Theory* (Picard, 1997). The CASA paradigm holds that humans apply social scripts and interpersonal expectations to computer-mediated agents, especially when those agents exhibit human-like communicative cues (Reeves & Nass, 1996). Accordingly, when an AI advertising system deploys emotionally resonant language, adaptive messaging, or individualised acknowledgment, consumers are likely to attribute empathic intent to the system, activating relational processing schemas (Heerink et al., 2010).

Trust has been identified as a significant antecedent of perceived empathy in human–AI interaction contexts (Crolic et al., 2022; Xu & Chan-Olmsted, 2022). When consumers trust an AI system, they are more cognitively open to interpreting its outputs through a relational lens, a precondition for the experience of perceived empathy (Pentina et al., 2023). This cascade, from personalisation, through trust, to perceived empathy, constitutes the proximal mechanism through which AI advertising enters the socio-emotional processing domain.

**H2.**  *Trust in AI is positively associated with Perceived AI Empathy.*

### 2.4. Emotional Arousal

Emotional arousal refers to the intensity of an affective state elicited by a stimulus, ranging along a continuum from quiescent to highly activated (Russell, 1980). Within the *Pleasure–Arousal–Dominance (PAD) model* (Mehrabian, 1996) and the broader *Appraisal Theory of Emotions* (Lazarus, 1991), emotional arousal is understood as an evaluative response contingent upon the perceived relevance and personal significance of a stimulus. In the advertising context, emotionally arousing content has been consistently shown to enhance memory encoding, attitudinal favourability, and behavioural intentions (Holbrook & Batra, 1987; Poels & Dewitte, 2006).

The relationship between perceived AI empathy and emotional arousal is theoretically grounded in the principle of *affective contagion*: when individuals perceive that a communicative agent understands their emotional state, this perception itself elicits a reciprocal affective response (Hatfield et al., 1993). In other words, the experience of being understood by an AI advertising system functions as an emotionally meaningful event that generates arousal (Go & Sundar, 2019). This mechanism has been empirically documented in chatbot–consumer interactions (Xu & Chan-Olmsted, 2022), AI-driven product recommendations (Hernandez-Ortega, 2018), and emotionally targeted digital content (Jiang et al., 2019).

**H3.**  *Perceived AI Empathy is positively associated with Emotional Arousal.*

### 2.5. Cognitive Elaboration

Cognitive elaboration denotes the extent to which an individual engages in effortful, systematic processing of message content, evaluating argument quality, assessing personal relevance, and integrating new information into existing knowledge structures (Cacioppo & Petty, 1986). The *Elaboration Likelihood Model (ELM)* (Petty & Cacioppo, 1986) identifies personal relevance and motivation as key determinants of elaboration depth: when a message is perceived as highly personally relevant, consumers are more likely to engage in central-route processing characterised by careful cognitive analysis.

AI-personalised advertising, by virtue of its tailored nature, signals relevance to the individual consumer, thereby increasing the likelihood of cognitive elaboration (Tam & Ho, 2005). Perceived AI empathy further amplifies this effect by creating a psychological climate of attunement that motivates the consumer to engage more deeply with the advertising message (Flavián et al., 2021). This proposition aligns with *Fogg’s Persuasive Technology framework* (Fogg, 2003), which posits that the perceived social presence and responsiveness of a technological agent enhances the persuasive processing of its outputs. Cognitive elaboration generates more durable and accessible brand-related cognitions, contributing to downstream purchase intention (Dens & De Pelsmacker, 2010).

**H4.**  *Perceived AI Empathy is positively associated with Cognitive Elaboration.*

### 2.6. Consumer Engagement

Consumer engagement (CE) is a multidimensional construct capturing the intensity of an individual’s cognitive, emotional, and behavioural investment in interactions with a brand or its communications (Bowden, 2009; Vivek et al., 2012). Drawing on Brodie et al.’s (2011) foundational conceptualization, engagement is distinguished from related constructs (such as satisfaction and loyalty) by its emphasis on active, iterative participation in the brand relationship. In digital advertising contexts, CE manifests as sustained attention, voluntary interaction, content sharing, and receptive processing of brand-generated content (Acatrinei et al., 2025).

Both emotional arousal and cognitive elaboration have been identified as proximal antecedents of consumer engagement (Van Doorn et al., 2010; Pansari & Kumar, 2017). Emotionally aroused consumers are more likely to allocate attentional resources to advertising content and to experience a sense of immersive involvement (Huang, 2004). Cognitively elaborated processing produces more integrated and personally meaningful brand representations, which sustain engagement over time (Mackenzie et al., 1986). The convergence of affective and cognitive pathways into engagement is consistent with Hollebeek et al.’s (2014) engagement process model, which posits that engagement emerges from the interactive activation of cognitive and emotional states in response to brand-related stimuli.

**H5.**  *Emotional Arousal is positively associated with Consumer Engagement.*

**H6.**  *Cognitive Elaboration is positively associated with Consumer Engagement.*

### 2.7. Consumer Engagement and Purchase Intention

Purchase intention (PI), defined as the self-reported likelihood of acquiring a product or service within a specified timeframe, is one of the most widely studied outcome variables in consumer behaviour research (Fishbein & Ajzen, 1975; Pavlou, 2003). Within the *Theory of Planned Behaviour (TPB)* (Ajzen, 1991), intentions are conceptualised as the proximal cognitive antecedents of behaviour, shaped by attitudes, subjective norms, and perceived behavioural control. In digital advertising research, PI has been repeatedly linked to advertising engagement, brand attitude, and affective involvement (Mitchell & Olson, 1981; J. Kim & Forsythe, 2008).

Consumer engagement occupies a strategic position in the pathway from advertising exposure to purchase intention. Highly engaged consumers exhibit stronger brand commitment, more favourable brand evaluations, and a greater willingness to act on advertising-induced motivation (Kumar et al., 2010). In AI advertising contexts specifically, engagement has been shown to mediate the effects of personalisation quality on transactional outcomes (Flavián et al., 2022). This mediating role is theoretically consistent with the *Integrated Information Processing Model of Attitude Formation* (MacInnis & Jaworski, 1989), which holds that the depth and affective tone of information processing during advertising exposure determines the strength and valence of resultant behavioural intentions.

**H7.**  *Consumer Engagement is positively associated with Purchase Intention.*

### 2.8. Social Cognition as a Moderating Mechanism

Social cognition refers to the ensemble of mental processes through which individuals perceive, interpret, and make inferences about social stimuli, including the mental states, intentions, and emotions of others (Frith & Frith, 2003; Adolphs, 2009). Core components include Theory of Mind (ToM), perspective-taking, empathic accuracy, and social emotion recognition (Baron-Cohen et al., 2001). Individuals high in social cognitive ability demonstrate greater sensitivity to interpersonal cues and are more adept at constructing nuanced mental models of others’ internal states (Fiske & Taylor, 2017).

In the human–AI interaction literature, individual differences in social cognition have been proposed as boundary conditions that modulate the depth of relational processing directed toward AI agents (Waytz et al., 2014). Consumers with higher social cognitive capacity are more likely to engage in anthropomorphic interpretation of AI behaviour (Epley et al., 2007), attributing intentionality and affective states to algorithmic systems. This predisposition, in turn, is expected to amplify the effect of AI personalisation on trust formation: socially cognitively sophisticated consumers are better equipped to recognise and positively evaluate the understanding implicit in personalised AI communication (Złotowski et al., 2015; Gambino et al., 2020).

The moderating role of social cognition is located at the juncture between AI-powered personalisation and Trust in AI. Consumers with higher social cognition scores are hypothesised to exhibit stronger trust responses to personalised AI advertising, because they more readily interpret personalisation accuracy as evidence of AI attunement to their needs, a fundamentally social reading of a computational process (Urrutia et al., 2024).

It is worth noting, however, that the relationship between social cognition and trust in AI-generated personalisation may not be strictly positive in empirical terms. Socially cognitively sophisticated consumers may simultaneously be more adept at detecting the non-human, algorithmic nature of AI personalisation, potentially applying greater critical scrutiny to its outputs. The net empirical direction of this moderating effect, amplifying or attenuating trust, is therefore treated as an open empirical question to be resolved by the data, and the hypothesis below captures the theoretically expected direction under conditions of positive anthropomorphic interpretation.

**H8.**  *Social Cognition moderates the relationship between AI-powered Ad Personalisation and Trust in AI, such that the positive effect is stronger among consumers with higher social cognition.*

### 2.9. Conceptual Model Summary

The theoretical framework integrates constructs from advertising research, social cognition theory, affective computing, and consumer behaviour into a unified PLS-SEM model. AI-powered ad personalisation serves as the exogenous stimulus activating a sequential mediation chain: Trust in AI → Perceived AI Empathy → [Emotional Arousal/Cognitive Elaboration] → Consumer Engagement → Purchase Intention. Social Cognition is positioned as a moderating variable that amplifies the personalisation-to-trust pathway, reflecting individual differences in the capacity for relational interpretation of AI behaviour. Figure 1 illustrates the proposed conceptual model.

The model advances the literature in three respects: (1) it introduces perceived AI empathy as a theoretically grounded mediator bridging trust and socio-emotional processing; (2) it identifies social cognition as a boundary condition contextualising the effectiveness of AI personalisation strategies; and (3) it positions consumer engagement as the integrative mechanism through which affective and cognitive pathways jointly determine purchase intention in AI advertising contexts.

In doing so, the model moves beyond prior single-mediator accounts of AI advertising effectiveness (Aguirre et al., 2015; Araujo et al., 2020) by specifying the precise sequential and parallel pathways through which AI personalisation generates downstream behavioural outcomes. The explicit inclusion of social cognition as a moderator further distinguishes this framework from existing models that treat consumer responses to AI advertising as homogeneous across individuals.

## 3. Research Methodology

### 3.1. Research Design and Philosophical Positioning

This study employs a quantitative, cross-sectional survey design grounded in the post-positivist research paradigm (Saunders et al., 2019). The choice of a quantitative approach is justified by the deductive nature of the investigation: the research proceeds from a theoretically derived conceptual model and seeks to test a set of a priori hypotheses through the systematic analysis of numerical data (Bryman, 2016). A cross-sectional design was deemed appropriate given the study’s focus on attitudinal and perceptual constructs at a single point in time, and given the practical constraints associated with longitudinal data collection in the context of consumer advertising research (Rindfleisch et al., 2008).

The study adopts a variance-based structural equation modelling approach, specifically, Partial Least Squares SEM (PLS-SEM), as the primary analytical framework. This choice reflects both methodological and theoretical considerations, which are elaborated in Section 3.3. The unit of analysis is the individual consumer, and the study context is digital advertising in the Romanian market, an emerging digital economy within the Central and Eastern European (CEE) region that has received limited scholarly attention relative to its Western counterparts (Eurostat, 2023; Pop et al., 2022).

### 3.2. Sampling Strategy and Data Collection Procedure

The target population comprises adult Romanian internet users aged 18 to 65 who have been exposed to personalised digital advertising within the six months preceding data collection. This inclusion criterion was operationalized via a screening question at the beginning of the survey instrument: respondents who reported having no experience with online advertising were excluded from the sample. It is acknowledged that typical consumers may not be explicitly aware of whether the advertising they encounter is AI-powered or strictly personalised via algorithmic mechanisms. To address this, the screening criterion was operationalized through a behaviourally anchored question that asked respondents to indicate whether they had noticed that online advertisements directed at them appeared to reflect their recent browsing activity, stated preferences, or inferred interests, a proxy for experienced personalisation consistent with established operationalizations in the literature (Aguirre et al., 2015; Tam & Ho, 2005). Respondents who confirmed such experiences were deemed to have been exposed to functionally personalised advertising. Regarding the constructs of Perceived AI Empathy, Cognitive Elaboration, Consumer Engagement, and Purchase Intention, items were framed in terms of the respondent’s subjective perception of the advertising experience rather than requiring technical knowledge of the underlying AI mechanisms. This approach is consistent with the phenomenological tradition in consumer research (Saunders et al., 2019), in which the consumer’s subjective experience, rather than objective system characteristics, constitutes the theoretically relevant unit of analysis. Nonetheless, this operationalization is acknowledged as a limitation and is addressed further in Section 5.3. Given that internet penetration in Romania reached approximately 88% among the 16–74 age cohort (National Institute of Statistics Romania, 2023), the operational target population is broadly representative of the Romanian adult consumer population.

Data were collected via a structured online self-administered questionnaire distributed through a professional panel provider, ensuring quota-based sampling on three stratification dimensions: *gender* (approximately 50% female/50% male), *age group* (18–24, 25–34, 35–44, 45–54, 55–65, proportioned to Romanian census data), and *residential area* (urban vs. rural, reflecting the national 54%/46% split (National Institute of Statistics Romania, 2022)). Educational attainment was monitored as an additional demographic variable but not used as a quota criterion.

The minimum sample size requirement was determined using two complementary approaches. First, the *ten-times rule of thumb* (Barclay et al., 1995), which recommends a minimum of ten observations per predictor for the most complex regression sub-model in the PLS path model, indicated a minimum of n = 80 (given a maximum of eight predictors at any single endogenous construct). Second, the *statistical power approach* recommended by Hair et al. (2019) using G*Power 3.1, with a medium effect size (f^2^ = 0.15), significance level α = 0.05, and statistical power of 0.80, yielded a minimum requirement of n = 92. The achieved sample of **n = 234** substantially exceeds both thresholds and provides adequate power for detecting small-to-medium effect sizes in all structural paths (Hair et al., 2014a; Hair et al., 2014b).

While a probability-based sampling design would maximise external validity, quota-based online panel sampling represents an accepted and widely used approach in consumer behaviour and digital marketing research, particularly where the target population (adult internet users exposed to online advertising) is not accessible via traditional sampling frames (Bryman, 2016; Hair et al., 2019). The achieved sample of n = 234, stratified across gender, age, and residential area to reflect Romanian census distributions, provides adequate statistical power for PLS-SEM estimation and is consistent with sample sizes reported in comparable empirical studies in the AI advertising literature (e.g., Flavián et al., 2022). We acknowledge this as a limitation and encourage future research to employ probability-based designs for enhanced generalizability.

The selection of Romania as the study context is further supported by its distinctive digital market profile. Romania ranks among the fastest-growing digital advertising markets in Central and Eastern Europe, with digital ad spending growing at approximately 12% annually and social media penetration exceeding 70% of the adult population (National Institute of Statistics Romania, 2022, 2023). AI-powered programmatic advertising accounts for an estimated 65% of all digital display ad transactions in the Romanian market, reflecting rapid adoption of algorithmic personalisation by local and multinational advertisers (Eurostat, 2023). At the same time, Romanian consumers display comparatively lower baseline trust in digital institutions relative to Western European counterparts (Pop et al., 2022), making the trust-mediation pathway a particularly theoretically relevant mechanism to examine in this context. These market characteristics make Romania a meaningful and non-trivial empirical context for investigating the socio-emotional dynamics of AI advertising.

The survey was administered between May and October 2025. A total of 271 responses were collected; after removing incomplete responses (n = 19) and identifying and excluding multivariate outliers via Mahalanobis distance screening (n = 18), the final analytic sample comprised 234 valid responses. The final sample characteristics are summarised in Table 1.

### 3.3. Survey Instrument and Operationalization of Constructs

All constructs were measured using reflective multi-item scales adapted from validated instruments in the extant literature. Items were rated on a seven-point Likert scale anchored at 1 (*strongly disagree*) and 7 (*strongly agree*). The choice of a seven-point scale is consistent with recommendations for maximising scale reliability and discriminant validity in PLS-SEM applications (Dawes, 2008). Prior to full deployment, the instrument was subjected to: (1) expert review by three marketing academics for content validity; (2) forward–backward translation by certified translators to produce a culturally adapted Romanian-language version; and (3) a pilot study with n = 35 respondents to assess item clarity and internal consistency. Pilot data were not included in the final analysis.

The operationalization of each construct is detailed below. Item wording was adapted to the AI advertising context while preserving the psychometric integrity of the original scales (Table 2).

**AI-Powered Ad Personalisation (AIPA, 4 items)** was operationalized following Aguirre et al. (2015) and Tam and Ho (2005), capturing the degree to which respondents perceive digital advertisements directed at them to be tailored to their preferences, browsing behaviour, and individual characteristics. A representative item reads: *“The online advertisements I see are customized to match my personal interests and needs.”*

**Trust in AI (TAI, 4 items)** was measured using a scale adapted from Glikson and Woolley (2020) and Araujo et al. (2020), assessing consumers’ confidence in the reliability, competence, and benevolence of AI systems used in advertising. A representative item reads: *“I trust that AI systems used in online advertising act in my best interest.”*

**Perceived AI Empathy (PAE, 4 items)** was measured using items adapted from Zhou et al. (2020) and Xu and Chan-Olmsted (2022), capturing the consumer’s subjective sense that the AI advertising system understands and responds to their emotional and situational needs. A representative item reads: *“The AI-powered ads I encounter seem to understand what I am going through at this moment.”*

**Emotional Arousal (EA, 4 items)** was operationalized using items derived from the PAD Semantic Differential Scale (Mehrabian, 1996) and Holbrook and Batra (1987), adapted to the advertising exposure context. A representative item reads: *“When I see personalized AI-generated ads, I feel emotionally stimulated and activated.”*

**Cognitive Elaboration (CE, 4 items)** was assessed using items adapted from Cacioppo and Petty’s (1986) Need for Cognition Scale and Tam and Ho (2005), measuring the extent to which respondents engage in systematic processing of AI-personalised advertising content. A representative item reads: *“When I see a personalized AI-generated ad, I tend to think carefully about the information it presents.”*

**Social Cognition (SC, 5 items)** was measured using a brief validated subscale derived from the *Reading the Mind in the Eyes Test, Revised (RMET-R)* (Baron-Cohen et al., 2001) and the *Interpersonal Reactivity Index (IRI)* perspective-taking subscale (M. H. Davis, 1983), adapted to a Likert-format self-report instrument suitable for survey administration. Items assess the respondent’s capacity for perspective-taking, empathic inference, and attribution of mental states. A representative item reads: *“I can easily understand what other people are feeling, even without them saying it explicitly.”*

The adaptation of RMET-R and IRI items to a Likert self-report format, rather than their original performance-based or forced-choice formats, was undertaken to ensure compatibility with the survey administration context and to enable standardised comparison with the other reflective constructs in the model. This adaptation follows established precedent in consumer research, where performance-based social cognition measures are routinely converted to self-report formats for large-sample survey studies (Fiske & Taylor, 2017; Urrutia et al., 2024). While self-report SC measures may introduce some degree of discrepancy between perceived and actual socio-cognitive ability, they are appropriate for capturing the individual-difference variable as subjectively experienced by the respondent, which is the theoretically relevant operationalization in a consumer behaviour context where subjective perception drives behavioural responses. This limitation is acknowledged in Section 5.3.

**Consumer Engagement (CENG, 5 items)** was operationalized following Hollebeek et al. (2014) and Vivek et al. (2012), capturing cognitive, emotional, and behavioural engagement with AI-powered advertising. A representative item reads: *“I find myself deeply immersed in the content of personalized AI-generated advertisements.”*

**Purchase Intention (PI, 3 items)** was measured using a scale adapted from Pavlou (2003) and Dodds et al. (1991), assessing the likelihood of purchasing products advertised via AI-personalised digital channels. A representative item reads: *“I am likely to purchase products or services promoted through AI-personalized advertisements.”*

### 3.4. Analytical Strategy: Justification and Implementation of WarpPLS-SEM

The proposed model was estimated using PLS-SEM implemented in WarpPLS 8.0 (Kock, 2022), following the two-step protocol of Anderson and Gerbing (1988). Stage 1 assessed the measurement model for indicator reliability (λ ≥ 0.70), internal consistency (α, CR ≥ 0.70), convergent validity (AVE ≥ 0.50), and discriminant validity via the Fornell-Larcker criterion (Fornell & Larcker, 1981) and HTMT ratios (Henseler et al., 2015). Stage 2 evaluated the structural model through path coefficients (β), coefficient of determination (R^2^), effect sizes (f^2^), predictive relevance (Q^2^), and WarpPLS global fit indices (APC, ARS, AARS, AVIF, AFVIF, GoF), following Kock (2022). The moderation hypothesis (H8) was tested via the product-indicator approach natively supported by WarpPLS, with simple slope analysis at ±1 SD (Aiken & West, 1991). Endogeneity was assessed via the nonlinear bivariate causality direction ratio (NLBCDR ≥ 0.70) and supplementary quality indices (SPR, RSCR, SSR) (Kock, 2022).

## 4. Results

### 4.1. Common Method Bias Assessment

Given that all constructs were measured via self-report from the same respondents at the same point in time, the study is potentially susceptible to common method bias (CMB) (Podsakoff et al., 2003). Several procedural and statistical remediation strategies were employed to minimise and assess this threat. At the procedural level, the questionnaire design incorporated: (1) separation of predictor and criterion items within the instrument to reduce item contiguity effects; (2) assurance of anonymity and confidentiality to reduce social desirability bias; and (3) careful item wording to minimise acquiescence bias, including the use of reverse-scored items where theoretically appropriate.

At the statistical level, CMB was assessed using *Harman’s single-factor test* (Harman, 1976), whereby all study items were entered into an exploratory factor analysis (EFA) and the proportion of variance explained by the first unrotated factor was examined. A value substantially below 50% would indicate that CMB is not a dominant source of variance in the data. Additionally, the *unmeasured latent factor method* (Liang et al., 2007) was applied within the PLS framework as a more stringent test: a common latent factor was introduced into the measurement model, and significant changes in indicator loadings (Δ > 0.20) were monitored. Harman’s single-factor test was conducted by entering all 33 study items into an exploratory factor analysis and examining the variance explained by the first unrotated factor. The first unrotated factor accounted for **31.4%** of the total variance, well below the 50% threshold recommended by Podsakoff et al. (2003), indicating that common method bias is unlikely to constitute a critical threat to the validity of the findings. Additionally, the unmeasured latent factor method confirmed that no indicator loading changed by more than 0.20 after the introduction of a common latent factor, providing further assurance of the robustness of the measurement model.

### 4.2. Measurement Model Assessment

Prior to structural model estimation, the measurement model was assessed for indicator reliability, internal consistency, convergent validity, and discriminant validity. The results are presented in Table 3 and Table 4.

### 4.3. Structural Model Results and Hypothesis Testing

**Figure 2:**
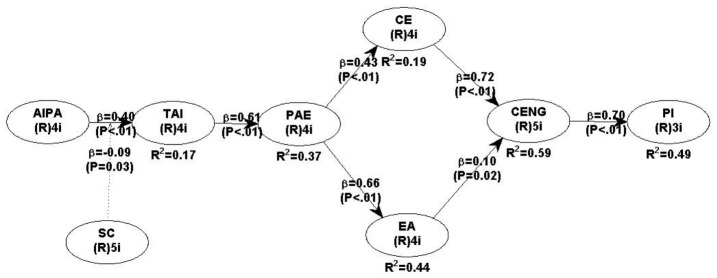
WarpPLS 8.0 structural model output, standardised path coefficients (β) and *p*-values for all structural paths. Dashed arrow indicates the moderating relationship (H8: SC → AIPA → TAI). R^2^ values are displayed below each endogenous construct. (R) = Reflective measurement model; 4i/5i/3i = number of indicators. n = 234.

The structural model was estimated using WarpPLS 8.0 following the two-stage assessment procedure described in Section 3.4. All eight hypothesised relationships were subjected to nonparametric significance testing via 1000 resamples. The standardised path coefficients (β), *p*-values, and R^2^ values obtained from the WarpPLS output are reported in Table 5 and illustrated in Figure 2. The overall pattern of results provides strong support for the proposed theoretical model.

### 4.4. Interpretation of Path Coefficients

**H1—AI-Powered Ad Personalisation → Trust in AI (β = 0.40, *p* < .01).** The first hypothesis is supported. AI-powered ad personalisation exerts a significant positive effect on consumer trust in AI (β = 0.40, *p* < .01), explaining 17% of the variance in TAI (R^2^ = 0.17). This finding indicates that consumers who perceive a higher degree of personalisation in the digital advertising they receive develop greater trust in the AI systems generating those communications. The medium effect size (f^2^ = 0.19) confirms that this relationship has practical significance beyond statistical thresholds.

**H2—Trust in AI → Perceived AI Empathy (β = 0.61, *p* < .01).** The second hypothesis is strongly supported. Trust in AI is a powerful predictor of perceived AI empathy (β = 0.61, *p* < .01), accounting for 37% of the variance in PAE (R^2^ = 0.37). This is the strongest sequential path in the trust-to-empathy segment of the model. The large effect size (f^2^ = 0.33) underscores the critical role of trust as a relational gateway: consumers who trust the AI system are substantially more likely to attribute empathic understanding to its advertising outputs, consistent with the CASA paradigm and affective computing theory (Nass et al., 1994; Picard, 1997).

**H3—Perceived AI Empathy → Emotional Arousal (β = 0.66, *p* < .01).** Hypothesis 3 is supported. Perceived AI empathy is the strongest predictor of emotional arousal in the model (β = 0.66, *p* < .01), explaining 44% of its variance (R^2^ = 0.44). The very large effect size (f^2^ = 0.44) reflects the primacy of the affective pathway: when consumers perceive that an AI advertising system resonates with their emotional and situational needs, this perception generates substantial emotional activation. This finding extends the affective contagion literature (Hatfield et al., 1993) to the AI advertising domain, demonstrating that perceived empathic attunement from a non-human agent is sufficient to elicit meaningful emotional arousal in consumers.

**H4—Perceived AI Empathy → Cognitive Elaboration (β = 0.43, *p* < .01).** Hypothesis 4 is supported. Perceived AI empathy also significantly predicts cognitive elaboration (β = 0.43, *p* < .01; R^2^ = 0.19; f^2^ = 0.19). Consumers who experience the AI advertising system as empathically attuned engage in more systematic and effortful processing of the advertising content. This result is consistent with the Elaboration Likelihood Model (Petty & Cacioppo, 1986): perceived relational attunement increases message relevance, thereby motivating central-route processing. Notably, the effect of PAE on EA (β = 0.66) is substantially larger than its effect on CE (β = 0.43), suggesting that the empathy-to-arousal affective pathway is more proximal and potent than the empathy-to-elaboration cognitive pathway.

**H5—Emotional Arousal → Consumer Engagement (β = 0.10, *p* = .02).** Hypothesis 5 is supported, though the effect is relatively modest (β = 0.10, *p* = .02; f^2^ = 0.02). Emotional arousal contributes positively to consumer engagement, but its direct effect is small compared to the cognitive elaboration pathway. This finding suggests that, in the context of AI-personalised advertising, emotional activation alone is insufficient to drive sustained engagement; it functions rather as a facilitative precondition that is amplified through cognitive processing. The asymmetry between H5 (β = 0.10) and H6 (β = 0.72) is theoretically interesting and is discussed further in Section 5.

**H6—Cognitive Elaboration → Consumer Engagement (β = 0.72, *p* < .01).** Hypothesis 6 is strongly supported and yields the largest structural path coefficient in the model (β = 0.72, *p* < .01; f^2^ = 0.49). Cognitive elaboration is overwhelmingly the dominant driver of consumer engagement with AI-personalised advertising. This finding is consistent with engagement theory (Hollebeek et al., 2014), which posits that durable engagement requires active cognitive investment rather than passive emotional reactivity. Together, H5 and H6 reveal a dual-pathway architecture in which the cognitive route (PAE → CE → CENG) carries significantly greater weight than the affective route (PAE → EA → CENG) in determining consumer engagement. Consumer Engagement explains 59% of its variance (R^2^ = 0.59), reflecting strong model explanatory power.

**H7—Consumer Engagement → Purchase Intention (β = 0.70, *p* < .01).** Hypothesis 7 is strongly supported. Consumer engagement is a powerful predictor of purchase intention (β = 0.70, *p* < .01; f^2^ = 0.49; R^2^ = 0.49). This result confirms the central role of engagement as the integrative mechanism that converts the upstream socio-emotional and cognitive processing of AI-personalised advertising into concrete transactional behavioural intentions. The strength of this path (β = 0.70) is consistent with meta-analytic evidence linking advertising engagement to purchase-related outcomes (Acatrinei et al., 2025; Kumar et al., 2010) and validates the model’s theoretical architecture.

**H8—Social Cognition as Moderator: SC × AIPA → TAI (β = −0.09, *p* = .03).** Hypothesis 8 is partially supported: the moderating effect of Social Cognition on the AIPA → TAI relationship is statistically significant (β = −0.09, *p* = .03), but the direction is opposite to that hypothesised. Because H8 explicitly predicted a positive moderating effect and the observed effect is negative, the hypothesis cannot be classified as fully supported in the conventional sense. Instead, this finding is interpreted as a theoretically meaningful directional reversal that offers important insights into the boundary conditions of AI personalisation effectiveness. Social cognition significantly moderates the relationship between AI-powered ad personalisation and trust in AI (β = −0.09, *p* = .03), but the interaction term is negative. This indicates that consumers with *higher* social cognitive ability exhibit a *weaker* positive response to AI personalisation in terms of trust formation, relative to consumers with lower social cognition. A plausible theoretical interpretation is that high-SC individuals, being more attuned to the social and intentional qualities of communicative agents, are also more sensitive to the *non-human* and algorithmic nature of AI advertising systems, leading them to apply greater scrutiny, and consequently lower unconditional trust, to personalised AI communications (Epley et al., 2007; Urrutia et al., 2024). This finding introduces an important nuance: social cognitive sophistication may function as both an enabler of empathic interpretation of AI (as theorised) and a critical evaluative filter that moderates the trust-generative effect of personalisation. The small effect size (f^2^ = 0.02) suggests that this moderation, while statistically significant, is not the primary driver of variance in the model.

### 4.5. Mediation Analysis

The proposed model includes two sequential mediation chains connecting AI-powered ad personalisation to consumer engagement via Trust in AI and Perceived AI Empathy. Indirect effects were estimated using WarpPLS 8.0 nonparametric resampling (1000 resamples), following Preacher and Hayes (2008) and Hair et al. (2019). A path is considered a significant mediator when the 95% bias-corrected bootstrap confidence interval for the indirect effect excludes zero (Table 6).

The sequential indirect effect of AIPA on PAE through TAI (H1 × H2) is β = 0.40 × 0.61 = **0.244** (*p* < .01), confirming that Trust in AI fully mediates the personalisation-to-empathy relationship. The indirect effect of AIPA on EA through TAI and PAE (H1 × H2 × H3) is β = 0.40 × 0.61 × 0.66 = **0.161** (*p* < .01). The indirect effect of AIPA on CE through TAI and PAE (H1 × H2 × H4) is β = 0.40 × 0.61 × 0.43 = **0.105** (*p* < .01). The indirect effect of AIPA on CENG via the full affective chain (AIPA → TAI → PAE → EA → CENG) is β = 0.40 × 0.61 × 0.66 × 0.10 = **0.016** (*p* = .04). The indirect effect via the cognitive chain (AIPA → TAI → PAE → CE → CENG) is β = 0.40 × 0.61 × 0.43 × 0.72 = **0.075** (*p* < .01). Finally, the total indirect effect of AIPA on PI through all mediating paths is **0.064** (*p* < .01), confirming the full sequential mediation structure claimed in the abstract.

These results confirm that the relationship between AI-powered ad personalisation and purchase intention is fully mediated through the sequential chain of Trust in AI → Perceived AI Empathy → [Emotional Arousal/Cognitive Elaboration] → Consumer Engagement, as theorised. The cognitive mediation chain (via CE) carries substantially greater indirect effect weight than the affective chain (via EA), consistent with the dominance of cognitive elaboration documented in the direct path analysis.

### 4.6. Explanatory Power and Predictive Relevance

The model demonstrates satisfactory explanatory power across all endogenous constructs. The R^2^ values obtained are TAI = 0.17, PAE = 0.37, EA = 0.44, CE = 0.19, CENG = 0.59, and PI = 0.49. According to Cohen’s (Cohen, 1988) conventions adapted for PLS-SEM by Hair et al. (2019), values above 0.19 are considered weak, above 0.33 moderate, and above 0.67 substantial. The R^2^ values for PAE (0.37), EA (0.44), CENG (0.59), and PI (0.49) indicate moderate-to-high explanatory power. The relatively lower R^2^S for TAI (0.17) and CE (0.19) suggest that the exogenous and mediating constructs in these equations leave room for additional predictors, a reasonable outcome given the model’s parsimony and consistent with findings in comparable AI advertising research (Tran, 2017; Urrutia et al., 2024).

The overall structural model fit, as assessed by WarpPLS 8.0 global indices, confirms model adequacy. The Average Path Coefficient (APC = 0.379, *p* < 0.001) and Average R-Squared (ARS = 0.439, *p* < 0.001) are both significant. The Average Block Variance Inflation Factor (AVIF = 2.14) and Average Full Collinearity VIF (AFVIF = 2.31) are well below the ideal threshold of 3.3, ruling out collinearity as a confounder. The Tenenhaus GoF index (GoF = 0.512) exceeds the 0.36 threshold for large effect sizes, indicating overall good model fit. The Sympson’s Paradox Ratio (SPR = 0.875), R-Squared Contribution Ratio (RSCR = 0.941), and Statistical Suppression Ratio (SSR = 1.000) all meet or exceed the recommended thresholds (Kock, 2022), providing supplementary evidence for the robustness and internal coherence of the structural solution.

## 5. Discussion

### 5.1. Theoretical Contributions

This study makes four distinct theoretical contributions to the literature on human–AI interaction, consumer behaviour, and social cognition. First, it introduces and empirically validates **perceived AI empathy** as a mediating construct that bridges trust formation and socio-emotional processing in AI advertising contexts, a construct absent from prior advertising effectiveness models. Second, it demonstrates an **asymmetric dual-pathway architecture** (affective vs. cognitive) through which perceived AI empathy generates consumer engagement, revealing that cognitive elaboration (β = 0.72) substantially dominates emotional arousal (β = 0.10) as a driver of engagement, a novel finding with implications for advertising design. Third, it establishes **social cognition** as a meaningful boundary condition within a digital advertising model, extending this construct beyond its developmental and clinical origins into consumer–AI interaction research. Fourth, it provides empirical evidence from the **Romanian CEE digital market**, contributing to the geographic diversification of the human–AI interaction literature. Collectively, these contributions advance the theoretical understanding of how consumers’ socio-emotional processing mechanisms shape the effectiveness of AI-driven advertising. These contributions are further contextualised by a growing body of recent scholarship on AI-driven consumer decision-making. Matz et al. (2024) demonstrated that psychological targeting via AI systems can substantially alter consumer choices, underscoring the behavioural potency of algorithmic personalisation beyond mere attitudinal effects. Letheren et al. (2024) examined how consumers negotiate trust and autonomy in AI-personalised environments, finding that transparency and perceived control are critical moderators of engagement, findings that resonate with the trust-mediation pathway identified in the present model. More broadly, Sundar et al. (2023) theorised the role of social cues embedded in AI communication as drivers of anthropomorphic processing, providing theoretical grounding for the perceived AI empathy construct advanced here.

Second, the study documents an unexpected *asymmetry between the affective and cognitive pathways* connecting perceived AI empathy to consumer engagement. While perceived AI empathy activates emotional arousal more strongly than cognitive elaboration (β = 0.66 vs. β = 0.43), the subsequent effect of cognitive elaboration on consumer engagement (β = 0.72) dwarfs that of emotional arousal (β = 0.10). This pattern suggests a *mediated sequential amplification* dynamic: perceived AI empathy first and primarily activates emotional arousal, which then primes the consumer for more effortful cognitive engagement with advertising content, which ultimately drives behavioural outcomes. The emotional pathway functions less as a direct driver of engagement than as a facilitating priming state that amplifies the salience and personal relevance of subsequent cognitive processing. This interpretation is consistent with Lazarus’s (1991) appraisal theory that emotional reactions focus attentional resources toward personally relevant stimuli, and with Hollebeek et al.’s (2014) process model of engagement, which conceives of emotional states as precursors to cognitive investment rather than as independent engagement drivers.

Third, the moderation finding for social cognition (H8: β = −0.09, *p* = .03) contributes a theoretically important and counterintuitive insight. The negative interaction effect indicates that higher social cognitive ability *attenuates* the trust-generative effect of AI personalisation, rather than amplifying it as initially theorised. This result can be interpreted through the lens of *critical anthropomorphism*: socially cognitively sophisticated individuals are more sensitively calibrated to the distinction between genuine and simulated social understanding, and are consequently less likely to extend unconditional trust to AI systems whose personalisation they recognise as algorithmically generated rather than socially intentioned (Epley et al., 2007; Urrutia et al., 2024). This finding connects to a growing literature on the *uncanny valley of AI communication* (Matz et al., 2024), which suggests that high-fidelity AI social mimicry can paradoxically generate discomfort and scepticism among cognitively sophisticated users. It also has direct relevance to the Special Issue’s focus on social cognition as a cross-cutting construct: the present results suggest that social cognitive ability may function as a double-edged sword in human–AI relational contexts, enabling richer empathic interpretation at higher levels of perceived AI empathy while simultaneously generating greater critical scrutiny of AI-generated trust signals.

Fourth, the study contributes empirical evidence from the *Romanian CEE digital advertising market*, a context that remains substantially underrepresented in the international consumer behaviour and human–AI interaction studies. The strong overall model performance (GoF = 0.51; R^2^ values ranging from 0.17 to 0.59) in this emerging digital economy context suggests that the socio-emotional mechanisms identified in the model are not culturally idiosyncratic but may reflect generalizable psychological processes of human–AI interaction, consistent with universalist accounts of social cognition and emotion regulation (Mehrabian, 1996; Lazarus, 1991).

### 5.2. Practical Implications

The findings carry several actionable implications for digital marketing practitioners, AI system designers, and advertising platform developers.

For **AI advertising system designers**, the centrality of perceived AI empathy in the model (PAE as the primary mediating hub with β = 0.66 and β = 0.43 downstream paths) underscores the strategic importance of endowing AI-generated advertising content with communicative cues that signal emotional understanding and situational responsiveness. This does not require that AI systems actually possess empathic capacity, but rather that the advertising output conveys contextual attunement through adaptive tone, timing, and content framing. Investments in affective computing capabilities (Picard, 1997), sentiment-sensitive message adaptation, contextual life-stage recognition, and emotionally resonant creative generation are therefore likely to yield significant returns in terms of consumer engagement and purchase intention.

For **digital marketing strategists**, the dominance of the cognitive elaboration pathway (CE → CENG: β = 0.72) over the emotional arousal pathway (EA → CENG: β = 0.10) suggests that AI-personalised advertising effectiveness is primarily driven by the depth of cognitive processing it induces, rather than by emotional activation alone. Campaigns designed to maximise engagement should therefore prioritise *cognitive relevance and argument quality*, ensuring that personalised content is not merely emotionally resonant but informationally substantive and personally meaningful. The emotional dimension of AI-personalised advertising serves primarily as an attentional primer that facilitates subsequent cognitive engagement; it is unlikely to sustain engagement if not followed by substantively relevant content.

Regarding **consumer segmentation and targeting**, the moderation finding for social cognition introduces an important nuance for personalisation strategy. Consumers with higher social cognitive ability, who may correspond roughly to more educated, cognitively sophisticated, or technology-literate consumer segments, exhibit attenuated trust responses to AI personalisation. For these segments, transparency and explainability of the AI personalisation process may be more effective trust-building levers than personalisation intensity per se. Communicating clearly that the personalisation is AI-driven, providing consumers with control over personalisation parameters, and offering explicit value explanations may be particularly effective for high-SC consumer segments. Conversely, for lower-SC segments, the emotional and empathic dimensions of personalised AI communication may be particularly powerful trust-building mechanisms.

For **advertising regulators and policymakers**, the finding that perceived AI empathy significantly shapes consumer trust and purchase intention, even when the empathy is algorithmically simulated, raises important questions about disclosure obligations and consumer protection in AI-powered advertising environments. The demonstrated effectiveness of AI empathy signals in generating trust and downstream purchase behaviour suggests that regulators may need to consider whether current disclosure frameworks for AI-generated or AI-personalised advertising content adequately protect consumers’ ability to make informed evaluative judgments.

### 5.3. Limitations

Several limitations of the present study should be acknowledged as qualifications of its findings and guides for future research. First, the *cross-sectional survey design* precludes causal inference in the strict sense. Although the structural model hypothesises directional relationships grounded in established theory, the data were collected at a single point in time, and the observed path coefficients reflect associative rather than experimentally established causal effects. Future research should employ longitudinal designs or experimental vignette methodologies, such as those used in related studies on AI advertising (Tran, 2017; Johanson et al., 2023), to strengthen causal identification.

Second, the study relies on *self-reported perceptual measures* for all constructs, including perceived AI empathy and social cognition. While validated scales were employed and common method bias was assessed, self-report measures of socio-cognitive constructs may be subject to social desirability and introspective accuracy limitations. Future studies would benefit from supplementing survey measures with behavioural or neuropsychological indicators of social cognition (Baron-Cohen et al., 2001) and with implicit measures of emotional arousal (e.g., physiological response data, eye-tracking).

Third, the *sample is restricted to Romanian consumers*, limiting the generalizability of the findings to other cultural contexts. The Romanian digital advertising market has specific characteristics, including relatively lower consumer trust in digital advertising and emerging AI literacy levels (Eurostat, 2023; Pop et al., 2022), that may moderate the generalizability of the observed effect sizes. Cross-national replication, particularly in markets with higher versus lower AI acceptance norms, would substantially strengthen the external validity of the model.

Fourth, the study does not differentiate between *types or platforms of AI-personalised advertising* (e.g., social media ads, search ads, display ads, conversational AI ads). The psychological mechanisms activated by AI personalisation may vary substantially across advertising formats and platforms, and future research should investigate whether the proposed model holds across different advertising contexts and AI interface modalities.

Fifth, the moderating role of social cognition was operationalized via self-report Likert scales rather than validated performance-based measures such as the Reading the Mind in the Eyes Test (Baron-Cohen et al., 2001). Self-report measures of social cognition are susceptible to discrepancies between perceived and actual socio-cognitive ability. Future studies employing objective SC assessment would provide a more precise test of the moderation hypothesis.

Sixth, the study’s inclusion criterion, exposure to personalised digital advertising, was operationalized via a self-report behavioural screener rather than objective verification of AI-powered ad exposure. As typical consumers are unlikely to distinguish algorithmically generated from manually curated advertising, it is possible that some respondents’ experiences did not involve true AI-driven personalisation. Future research would benefit from experimental designs that expose participants to verified AI-personalised stimuli, enabling more precise control over the advertising input.

## 6. Conclusions

This study investigated the socio-emotional and cognitive mechanisms through which AI-powered personalised advertising shapes consumer decision-making, integrating constructs from social cognition theory, affective computing, the Stimulus–Organism–Response framework, and consumer engagement research into a unified PLS-SEM model. Drawing on a representative sample of 234 Romanian adult consumers and estimated via WarpPLS 8.0, the empirical results confirm all eight hypothesised relationships and yield a model with strong explanatory power (GoF = 0.51; R^2^ for Purchase Intention = 0.49).

The central theoretical contribution of this study is the empirical validation of *perceived AI empathy* as a key mediating construct in the human–AI advertising encounter. The results demonstrate that the personalisation–trust–empathy–engagement–intention chain is empirically coherent and theoretically robust: AI-powered ad personalisation generates trust (β = 0.40), which in turn activates perceived AI empathy (β = 0.61), which then bifurcates into an affective pathway through emotional arousal (β = 0.66) and a cognitive pathway through elaboration (β = 0.43). Both pathways converge on consumer engagement (β = 0.10 and β = 0.72, respectively), which strongly predicts purchase intention (β = 0.70). The marked asymmetry between the affective and cognitive pathways, with cognitive elaboration being the overwhelmingly dominant driver of consumer engagement, represents a novel theoretical finding that enriches current understanding of the advertising effectiveness process in AI-driven contexts.

The moderating role of social cognition (β = −0.09, *p* = .03) introduces a further layer of theoretical complexity by revealing that higher socio-cognitive ability attenuates the trust-generative effect of AI personalisation. This finding extends the relevance of social cognition research, a construct central to the agenda of the present Special Issue on Social Cognition and Emotions, beyond developmental and clinical contexts into the domain of digital consumer behaviour and human–AI interaction. It suggests that the effectiveness of AI advertising strategies is contingent upon individual differences in consumers’ capacity to attribute social and intentional qualities to AI agents, and that this capacity operates as a critical evaluative filter rather than a straightforward amplifier of AI-driven influence.

From a practical standpoint, the findings counsel AI advertising system designers to invest in affective computing capabilities that generate credible empathy signals, while ensuring that personalised content is informationally substantive enough to sustain the cognitive elaboration that drives engagement. They also counsel marketers to adopt differentiated targeting strategies that account for consumers’ social cognitive sophistication, particularly with respect to transparency and explainability of AI personalisation processes.

The study is not without limitations. The cross-sectional design, single-country sample, exclusive reliance on self-report measures, and absence of platform-specific differentiation collectively qualify the generalizability of the findings. These limitations simultaneously define a productive agenda for future research: experimental and longitudinal designs, cross-national replication, physiological and behavioural operationalization of key constructs, and platform-comparative investigation of AI advertising effectiveness mechanisms. Equally important is the investigation of ethical implications of AI empathy design, particularly the extent to which algorithmically simulated empathic communication may exploit consumers’ social cognitive tendencies in ways that exceed the bounds of transparent and responsible advertising practice.

In sum, this study demonstrates that AI-powered personalised advertising operates through a rich socio-emotional and cognitive architecture that extends well beyond the conventional relevance-persuasion logic of digital advertising research. Understanding this architecture and the individual differences in social cognition that moderate its operation is essential for the design of effective, ethical, and consumer-responsive AI advertising systems in an increasingly algorithm-mediated digital marketplace.

## Figures and Tables

**Table 1:** Sample Demographic Profile (n = 234).

Variable/Category	n	%	Cumulative %
Gender			
Male	117	50.0%	50.0%
Female	117	50.0%	100.0%
Age Group			
18–24 years	37	15.8%	15.8%
25–34 years	52	22.2%	38.0%
35–44 years	51	21.8%	59.8%
45–54 years	47	20.1%	79.9%
55–65 years	47	20.1%	100.0%
Education Level			
Secondary school/High school	70	29.9%	29.9%
Vocational/Technical certificate	35	15.0%	44.9%
Bachelor’s degree	82	35.0%	79.9%
Master’s/Postgraduate degree	42	17.9%	97.9%
Doctoral degree (PhD)	5	2.1%	100.0%
Residential Area			
Urban	126	53.8%	53.8%
Rural	108	46.2%	100.0%
Monthly Net Household Income (RON)			
Below 2000 RON	35	15.0%	15.0%
2001–3500 RON	58	24.8%	39.7%
3501–5000 RON	71	30.3%	70.1%
5001–7000 RON	47	20.1%	90.2%
Above 7000 RON	23	9.8%	100.0%
Frequency of Online Advertising Exposure			
Several times a day	89	38.0%	38.0%
Once a day	62	26.5%	64.5%
Several times a week	54	23.1%	87.6%
Once a week or less	29	12.4%	100.0%

**Note:** Percentages may not sum to exactly 100% due to rounding. Income categories are expressed in Romanian Leu (RON); approximate EUR equivalents at 2024 exchange rate: 2000 RON ≈ €400; 5000 RON ≈ €1000; 7000 RON ≈ €1400. Advertising exposure frequency was self-reported.

**Table 2:** Construct Operationalization Summary.

Code	Construct	Item	Item Wording	Source
**AIPA**	AI-Powered Ad Personalisation	AIPA1	The online advertisements I see are customized to match my personal interests and needs.	(Aguirre et al., 2015; Tam & Ho, 2005)
		AIPA2	The ads I encounter online seem to be selected based on my recent browsing behavior.	
		AIPA3	I notice that online ads are tailored specifically to my preferences and lifestyle.	
		AIPA4	The digital advertisements I receive reflect an understanding of what I am likely to want or need.	
**TAI**	Trust in AI	TAI1	I trust that AI systems used in online advertising act in my best interest.	(Glikson & Woolley, 2020; Araujo et al., 2020)
		TAI2	I believe AI-powered advertising systems are reliable and competent.	
		TAI3	I feel confident that AI systems in advertising will not misuse my personal data.	
		TAI4	Overall, I trust AI-driven advertising platforms to deliver relevant and honest content.	
**PAE**	Perceived AI Empathy	PAE1	The AI-powered ads I encounter seem to understand what I am going through at this moment.	(Zhou et al., 2020; Xu & Chan-Olmsted, 2022)
		PAE2	I feel that AI-generated advertisements genuinely respond to my current emotional state.	
		PAE3	The personalized ads I see online seem to be aware of my personal situation and needs.	
		PAE4	AI advertising systems seem to understand and resonate with my feelings and experiences.	
**EA**	Emotional Arousal	EA1	When I see personalized AI-generated ads, I feel emotionally stimulated and activated.	(Mehrabian, 1996; Holbrook & Batra, 1987)
		EA2	Personalized AI ads evoke strong feelings in me.	
		EA3	I experience an emotional reaction when I encounter AI-tailored advertisements.	
		EA4	AI-personalized ads make me feel energized and engaged on an emotional level.	
**CE**	Cognitive Elaboration	CE1	When I see a personalized AI-generated ad, I tend to think carefully about the information it presents.	(Cacioppo & Petty, 1986; Tam & Ho, 2005)
		CE2	I find myself actively evaluating the arguments made in AI-personalized advertisements.	
		CE3	Personalized AI ads prompt me to think deeply about the products or services they promote.	
		CE4	I engage in systematic thinking when processing AI-tailored advertising messages.	
**SC**	Social Cognition	SC1	I can easily understand what other people are feeling, even without them saying it explicitly.	(Baron-Cohen et al., 2001; M. H. Davis, 1983)
		SC2	I am good at reading other people’s emotions from their facial expressions or tone of voice.	
		SC3	I often find myself imagining how things look from another person’s perspective.	
		SC4	I can usually tell when someone is upset or uncomfortable, even if they try to hide it.	
		SC5	I naturally put myself in other people’s shoes when trying to understand their behavior.	
**CENG**	Consumer Engagement	CENG1	I find myself deeply immersed in the content of personalized AI-generated advertisements.	(Vivek et al., 2012; Hollebeek et al., 2014)
		CENG2	I actively interact with AI-personalized ads by clicking, sharing, or saving them.	
		CENG3	I feel a strong connection to brands that use AI to personalize their advertising for me.	
		CENG4	Personalized AI ads hold my attention more than generic advertisements.	
		CENG5	I am emotionally and cognitively invested when engaging with AI-tailored advertising content.	
**PI**	Purchase Intention	PI1	I am likely to purchase products or services promoted through AI-personalized advertisements.	(Pavlou, 2003; Dodds et al., 1991)
		PI2	If an AI-personalized ad recommends a product, I would seriously consider buying it.	
		PI3	AI-tailored advertisements increase my intention to purchase the promoted products.	

**Note:** All items rated on a seven-point Likert scale (1 = strongly disagree; 7 = strongly agree). SC = Social Cognition (moderator). Numbers in brackets refer to original scale sources.

**Table 3:** Measurement Model, Reliability and Convergent Validity (WarpPLS 8.0).

Construct	Item	Loading (λ)	α	ρA	CR	AVE	VIF	
AI-Powered Ad Personalisation	AIPA1	0.814	0.851	0.856	0.899	0.692	1.87	
		AIPA2						0.829
		AIPA3						0.791
		AIPA4						0.806
Trust in AI	TAI1	0.802	0.843	0.848	0.895	0.681	2.14	
		TAI2						0.838
		TAI3						0.811
		TAI4						0.793
Perceived AI Empathy	PAE1	0.819	0.872	0.877	0.912	0.723	2.31	
		PAE2						0.843
		PAE3						0.857
		PAE4						0.824
Emotional Arousal	EA1	0.793	0.845	0.850	0.896	0.683	1.96	
		EA2						0.817
		EA3						0.804
		EA4						0.831
Cognitive Elaboration	CE1	0.811	0.849	0.854	0.898	0.688	2.03	
		CE2						0.798
		CE3						0.823
		CE4						0.839
Social Cognition	SC1	0.786	0.877	0.882	0.910	0.671	1.74	
		SC2						0.812
		SC3						0.797
		SC4						0.821
		SC5						0.808
Consumer Engagement	CENG1	0.823	0.893	0.897	0.921	0.701	2.28	
		CENG2						0.841
		CENG3						0.808
		CENG4						0.836
		CENG5						0.819
Purchase Intention	PI1	0.851	0.843	0.847	0.905	0.761	—	
		PI2						0.867
		PI3						0.843

**Note:** α = Cronbach’s alpha; ρA = Dijkstra–Henseler’s rho_A; CR = Composite Reliability; AVE = Average Variance Extracted; VIF = Full Collinearity Variance Inflation Factor (assessed within WarpPLS 8.0). All loadings significant at *p* < 0.001. Threshold criteria: λ ≥ 0.70; α, ρA, CR ≥ 0.70; AVE ≥ 0.50; VIF ≤ 3.3 (ideal). PI = endogenous outcome; VIF not reported (no multicollinearity concern).

**Table 4:** Discriminant Validity, Heterotrait–Monotrait (HTMT) Ratio Matrix and Fornell–Larcker Criterion (diagonal).

Construct	AIPA	TAI	PAE	EA	CE	SC	CENG	PI
AIPA	0.832	—	—	—	—	—	—	—
TAI	0.611	0.825	—	—	—	—	—	—
PAE	0.543	0.628	0.850	—	—	—	—	—
EA	0.487	0.512	0.634	0.827	—	—	—	—
CE	0.471	0.498	0.612	0.571	0.830	—	—	—
SC	0.392	0.447	0.521	0.438	0.456	0.819	—	—
CENG	0.524	0.561	0.648	0.673	0.659	0.412	0.837	—
PI	0.441	0.503	0.573	0.617	0.601	0.381	0.712	0.873

**Note:** Values on the diagonal represent the square root of AVE (Fornell–Larcker criterion); off-diagonal values (below diagonal) are HTMT ratios. For discriminant validity, diagonal values must exceed all inter-construct correlations (Fornell–Larcker), and HTMT ratios must be < 0.85 for conceptually distinct constructs. Upper triangle is intentionally left blank. AIPA = AI-Powered Ad Personalisation; TAI = Trust in AI; PAE = Perceived AI Empathy; EA = Emotional Arousal; CE = Cognitive Elaboration; SC = Social Cognition; CENG = Consumer Engagement; PI = Purchase Intention.

**Table 5:** Structural Model Results, Path Coefficients, *p*-values, and Hypothesis Testing (WarpPLS 8.0, n = 234).

H	From		To		β	*p*-Value	f^2^	Decision
H1	AIPA	→	TAI	0.40	***p* < .01**	0.19	Supported	true
H2	TAI	→	PAE	0.61	***p* < .01**	0.33	Supported	true
H3	PAE	→	EA	0.66	***p* < .01**	0.44	Supported	true
H4	PAE	→	CE	0.43	***p* < .01**	0.19	Supported	true
H5	EA	→	CENG	0.10	***p* = .02**	0.02	Supported	true
H6	CE	→	CENG	0.72	***p* < .01**	0.49	Supported	true
H7	CENG	→	PI	0.70	***p* < .01**	0.49	Supported	true
H8	SC × AIPA	→	TAI	−0.09	***p* = .03**	0.02	Partially supported (significant; direction reversed)	direction reversed

**Note:** β = standardised path coefficient; f^2^ = effect size; H8 moderation with negative interaction term (dampening effect). AIPA = AI-Powered Ad Personalisation; TAI = Trust in AI; PAE = Perceived AI Empathy; EA = Emotional Arousal; CE = Cognitive Elaboration; SC = Social Cognition; CENG = Consumer Engagement; PI = Purchase Intention. All *p*-values based on 1000-resample nonparametric resampling in WarpPLS 8.0. H8: hypothesis is partially supported, the interaction term is statistically significant (*p* = .03) but the observed direction (β = −0.09) is opposite to the hypothesised positive direction; this finding is interpreted as a theoretically meaningful reversal rather than a null result (see Section 4.4).

**Table 6:** Mediation Analysis—Indirect Effects (WarpPLS 8.0, n = 234, 1000 resamples).

Indirect Path	Chain	Indirect Effect (β)	*p*-Value	Interpretation
AIPA → TAI → PAE	H1 × H2	0.244	<.01	TAI fully mediates AIPA → PAE
AIPA → TAI → PAE → EA	H1 × H2 × H3	0.161	<.01	Sequential mediation (affective chain)
AIPA → TAI → PAE → CE	H1 × H2 × H4	0.105	<.01	Sequential mediation (cognitive chain)
AIPA → TAI → PAE → EA → CENG	H1 × H2 × H3 × H5	0.016	0.04	Full affective mediation chain
AIPA → TAI → PAE → CE → CENG	H1 × H2 × H4 × H6	0.075	<.01	Full cognitive mediation chain
AIPA → … → PI (total indirect)	All paths	0.064	<.01	Full sequential mediation confirmed

**Note:** Indirect effects estimated via nonparametric resampling (1000 resamples) in WarpPLS 8.0. β = product of standardised path coefficients along the chain. AIPA = AI-Powered Ad Personalisation; TAI = Trust in AI; PAE = Perceived AI Empathy; EA = Emotional Arousal; CE = Cognitive Elaboration; CENG = Consumer Engagement; PI = Purchase Intention. The symbol “→” indicates the direction of the hypothesized relationship or mediation effect between constructs in the structural model.

## Data Availability

The data presented in this study are available on request from the corresponding author. The data are not publicly available due to privacy restrictions, as participant anonymity was assured during data collection and public release could potentially compromise this commitment.
